# 12q14 Microdeletions: Additional Case Series with Confirmation of a Macrocephaly Region

**DOI:** 10.1155/2015/192071

**Published:** 2015-07-22

**Authors:** Adrian Mc Cormack, Cynthia Sharpe, Nerine Gregersen, Warwick Smith, Ian Hayes, Alice M. George, Donald R. Love

**Affiliations:** ^1^Diagnostic Genetics, LabPLUS, Auckland City Hospital, P.O. Box 110031, Auckland 1148, New Zealand; ^2^Department of Neuroservices, Starship Children's Health, Private Bag 92024, Auckland 1142, New Zealand; ^3^Genetic Health Service New Zealand-Northern Hub, Auckland City Hospital, Private Bag 92024, Auckland 1142, New Zealand; ^4^Middlemore Hospital, Private Bag 93311, Otahuhu, Auckland 1640, New Zealand

## Abstract

To date, there have been only a few reports of patients carrying a microdeletion in chromosome 12q14. These patients usually present with pre- and postnatal growth retardation, and developmental delay. Here we report on two additional patients with both genotype and phenotype differences. Similar to previously published cases, one patient has haploinsufficiency of the *HMGA2* gene and shows severe short stature and developmental delay. The second patient is only one of a handful without the loss of the *HMGA2* gene and shows a much better growth profile, but with absolute macrocephaly. This patient's deletion is unique and hence defines a likely macrocephaly locus that contributes to the general phenotype characterising the 12q14 syndrome.

## 1. Introduction

Microarray technology has revolutionised the detection of human chromosomal abnormalities within cytogenetics by discovering new, as well as refining, existing syndromes. The technology has recently revealed the existence of a previously unknown deletion syndrome at 12q14. Initial case series have helped categorise and partially refine the genotype and phenotype correlations of this emerging syndrome [1–5], Table 1.

Menten et al. [2] investigated the causative gene for Osteopoikilosis using microarray technology and were the first to report three unrelated patients with* de novo* deletions of 12q14. Patients carrying heterozygous deletions in this region exhibited a common phenotype that included failure to thrive, short stature, and learning difficulties, which was noted to be phenotypically similar to Silver-Russell Syndrome (SRS). SRS is characterized by a variable clinical spectrum of pre- and postnatal growth retardation, relative macrocephaly, body asymmetry, and triangle facial gestalt [6, 7]. It is mainly caused by two mechanisms: maternal UPD chromosome 7 or hypomethylation at the imprinting centre region 1 (ICR1) on 11p15 [6, 7]. Generally, patients who fulfil specific clinical criteria can be reliably diagnosed with SRS; however, for 50% of patients the etiology is unknown and these patients are often referred to as having an “SRS-like” phenotype [7]. Of these patients, those with deletions in the 12q14 region form a subgroup.

The 12q14 interstitial deletions found in these initial cases encompassed two critical genes:* LEMD3* and* HMGA2*. Mutations in the former gene are implicated in Osteopoikilosis, while mutations in the latter gene in mice have a strong influence on height [8, 9]. Mari et al. [5] confirmed the role of* HMGA2* haploinsufficiency in the etiology of short stature and failure to thrive in reporting patients with deletions of this gene. A further case series reported by Buysse et al. [1] described patients without Osteopoikilosis, despite the loss of* LEMD3* gene, and confirmed that heterozygous intragenic deletions of* HMGA2* have a significant effect on human height. They proposed a minimum region of overlap encompassing approximately 11 genes that were thought to play a role in patients manifesting 12q14 microdeletion syndrome. Finally Takenouchi et al. [4] examined the correlation between the location (and extent) of deletions in 12q14 and clinical phenotype and proposed that relative macrocephaly may involve deletions upstream of the* HMGA2* gene.

The study described here comprises an additional two patients with interstitial 12q14 microdeletions. Patient 1 carries a 3.8 Mb heterozygous deletion involving the interstitial region 12q14.2q14.3, and Patient 2 carries a 5.6 Mb heterozygous deletion involving the interstitial region 12q14.1q14.3.

## 2. Clinical Report

### 2.1. Patient 1

A healthy male was born at 41-week gestation with asymmetrical growth restriction weighing 2550 g (<3rd centile), with length 48 cm (20th centile) and head circumference 35 cm (50th centile). The mother is a hyperactive, verbally aggressive and difficult individual who drank excessively during the pregnancy. She is also relatively short with a height of approximately 153 cm and has a strabismus. The father is approximately 183 cm tall, cannot read or write, and is socially reclusive. There were no concerns about the child's motor skills and achieved expected milestones. He was noted to be a behaviourally difficult child, always on the go, demanding attention, and seemingly always talking. He understood language and could talk well although at times his speech was unclear and he spoke in an unusual voice.

Paediatric assessment of the proband at three years of age noted that he was short, with height 82.6 cm (3.5 SD below the mean), weight 11 kg (0.5 kg less than the 3rd percentile), and head circumference 49.5 cm (on the 40th percentile). He had deep set eyes and a thin upper lip but normal philtrum and palpebral fissures each 22 mm (−2 SD). He wore glasses suggesting severe myopia and manifested alternating esotropia. He had no clear features of Fetal Alcohol Syndrome, but there were concerns over possible mild dysmorphic features. It was noted that social communication was poor and that he talked in unusual voice prosody, with reasonable receptive and expressive language. He was diagnosed with mild Autism Spectrum Disorder (ASD), was hyperactive and aggressive, and had many features of ADHD. A final genetics consultation at four years of age confirmed previous findings and his guardians stated that he had problems with fine motor skills. Head circumference was near the 50th centile and height below the 3rd centile. He had the appearance of relative macrocephaly but little else to see from a dysmorphology perspective. The child never had X-rays so we cannot say if there were skeletal changes consistent with a diagnosis of Osteopoikilosis.

### 2.2. Patient 2

A female was delivered at 35-week gestation following a breech presentation. Family medical history was unremarkable. Birth head circumference was 33 cm (75th–91st percentile for 35-week gestation), birth weight was 2.6 kg (75th percentile), and she had some issues with poor feeding. By 2 months of age her head had grown to 38.5 cm on the 98th percentile for her corrected gestational age and accelerated further to the 99th percentile by 6 months of age.

She passed her developmental screen at one year of age but a further assessment at 19 months revealed developmental delay. Macrocephaly was also first noted at this visit. Her head circumference has been stable 1-2 cm above the 98th percentile since then. Her motor skills were delayed and speech was also delayed and she met the criteria for ASD. She received speech therapy for six months, but this was stopped because at three years of age she had hundreds of words, was speaking in 2-3-word phrases, and understood most of what was said.

An MRI was performed at 27 months of age showing mild hyperintensity in the deep white matter in the periventricular regions of the occipital area bilaterally, more so on the right. There was no mass effect and the brain was normally formed, with no hydrocephalus noted.

**Figure 1:**
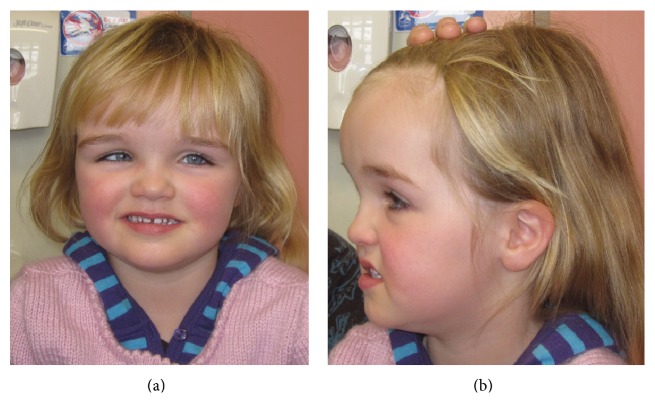
(a) Image of Patient 2. (b) Patient 2 exhibits frontal bossing and macrocephaly.

Further paediatric examination at 3 years 8 months of age showed height of 102.2 cm (75th centile), weight of 17.4 kg (75–90th centile), and head circumference of 54 cm (+3.5 SD). There were no obvious dysmorphic features or neurocutaneous stigmata and she was undergoing treatment for a strabismus. She had evidence of both gross motor/fine motor and language delay but was showing excellent catching up of her development with appropriate inputs. Her last review at 4 years 5 months showed that her head circumference continued to track above the centiles at 55 cm; she was 108 cm tall (75–90th centile) and weighed 20 kg (90th centile). She still met the criteria for a diagnosis of ASD and had global gross motor/fine motor and language delay but was showing very encouraging improvements in all aspects of her development. It was noted that she had mild plagiocephaly, a prominent brow, and mild hypertelorism (Figure 1(b)). The patient does not exhibit Osteopoikilosis, but she may be too young to manifest symptoms. The patient proved negative for mutations in the* PTEN* gene (by both dosage and sequencing), which was performed in light of macrocephaly and the diagnosis of autism.

### 2.3. Molecular Studies

**Figure 2:**
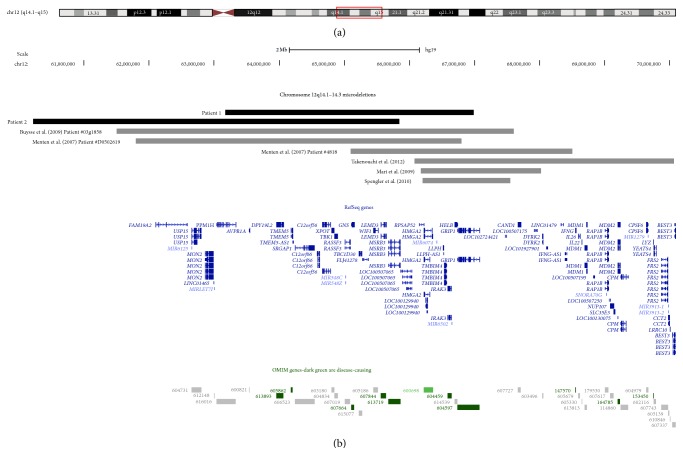
Schematic of chromosome 12q14 containing microdeletions. (a) shows an ideogram of chromosome 12. (b) shows the location and extent of the deletions detected in the patients described here and other cases reported in the literature, as well as RefSeq and OMIM genes that lie within 12q14. These graphics were taken from the UCSC genome browser (http://genome.ucsc.edu/).

DNA was extracted from both Patients 1 and 2 with genome-wide copy number analysis determined using an Affymetrix Cytogenetics Whole-Genome 2.7M array and CytoScan 750K Array, respectively, according to the manufacturer's instructions. Regions of copy number change were determined using the Affymetrix Chromosome Analysis Suite software (ChAS) either v.1.0.1 or v.1.2.2 and interpreted with the aid of the UCSC genome browser (http://genome.ucsc.edu/; Human March 2006 (hg18) assembly or February 2009 GRCh37/hg19 assembly), Figure 2.

Patient 1 carried a 3.8 Mb heterozygous deletion involving the interstitial chromosome region 12q14.2q14.3 (hg19 coordinates 63,169,460–66,983,229) encompassing 16 OMIM genes (*AVPR1A*,* DPY19L2*,* TMEM5*,* SRGAP1*,* XPOT*,* TBK1*,* RASSF3*,* GNS*,* TBC13D0*,* WIF1*,* LEMD3*,* MSRB3*,* HMGA2*,* IRAK3*,* HELB,* and part of* GRIP1*). Patient 2 carried a 5.6 Mb heterozygous deletion involving the interstitial chromosome region 12q14.1q14.3 (hg19 coordinates 60,220,054–65,843,601) encompassing 14 OMIM genes (*USP15*,* MIRLET7I*,* AVPR1A*,* DPY19L2*,* TMEM5*,* SRGAP1*,* XPOT*,* TBK1*,* RASSF3*,* GNS*,* TBC1D30*,* WIF1*,* LEMD3,* and part of* MSRB3*). The parents of Patient 1 were unavailable for follow-up analysis while the parents of Patient 2 showed normal molecular karyotypes.

## 3. Discussion

In the study described here, we report two patients with overlapping deletions in 12q14: a 3.8 Mb deletion in Patient 1 with extreme short stature, mild autism, behavioural problems, severe myopia, and esotropia and a 5.6 Mb deletion in Patient 2 with absolute macrocephaly, developmental delay, learning difficulties, and autism. The deletion in Patient 1 includes both the* LEMD3* and* HMGA2* genes, while the deletion in Patient 2 includes the* LEMD3* gene but not the* HMGA2* gene. The mother of Patient 1, who has short stature, behavioural issues, and a convergent strabismus, may also harbour a deletion, but unfortunately she was not available for testing to confirm this. Patient 2 had a* de novo* deletion.

Of the reported 12q14 interstitial deletion cases, only 5 (including one of the cases presented here) have not included the* HMGA2* gene. The deleted region detected in Patient 1 includes the* HMGA2* gene and this patient shows prenatal IUGR and severe short stature but critically the deleted region in Patient 2 does not encompass the* HMGA2* gene and they exhibit no pre- or postnatal growth issues. Both cases reinforce the importance of the* HMGA2* gene's influence on growth.

The* HMGA2* gene (OMIM 600698) encodes a mammalian high mobility group (HMG) protein which is implicated in transcriptional regulation [24]. Ligon et al. [25] described a* de novo* pericentric inversion in an 8-year-old boy causing an intragenic rearrangement which truncated the* HMGA2* gene, altering its expression and causing a phenotype which included overgrowth and lipomas. Interestingly,* hmga2* −/− mice show a “pygmy” phenotype with short stature and reduction in body fat [9]. Buysse et al. [1] reported a small intragenic deletion of the* HMGA2* gene in a boy with proportionate short stature, segregating within a larger pedigree with reduced adult height. In addition, Mari et al. [5] described a patient with a 1.84 Mb deletion encompassing six genes including the* HMGA2* gene in which the phenotype comprised pre- and postnatal growth restrictions and short stature.

Critically, Spengler et al. [3] proposed a separate locus distinct from* HMGA2* as being partially responsible for the SRS-like phenotype. Takenouchi et al. [4] reviewed deletions at 12q14 and showed that only a small group of patients had relative macrocephaly and short stature. They proposed two discernible groups of phenotypes of 12q14 microdeletions: a group of patients with short stature with relative macrocephaly and a group with an SRS-like phenotype and short stature without macrocephaly. They suggested a presumptive interval for relative macrocephaly spanning 0.5 Mb–2 Mb, but not including the* HMGA2* gene.

Patient 1, whose deletion includes the* HMGA2* gene, appears to have an SRS-like phenotype. Interestingly, Patient 2 lacks a growth retardation phenotype but has absolute macrocephaly confirming the suggestion made by Takenouchi et al. [4] of a macrocephaly locus that contributes to the SRS-like phenotype for patients with deletions in 12q14. At this time, the function of only some of the genes located in the deleted interval detected in Patient 2 has been characterised, Table 2.

Our cases, together with those reported earlier, suggest that the SRS-like phenotype of patients with deletions in 12q14 can be refined to the following:

1. Deletions that include (all or some of) the* HMGA2* gene correlate with a growth-based phenotype.
2. A macrocephaly region (~2 MB in length) proximal and independent of the* HMGA2* gene correlates with an absolute macrocephaly phenotype (see Patient 2).
3. Deletions involving both of these regions combine to form an SRS-like phenotype (see Patient 1).

In the future, smaller deletions within 12q14 may be uncovered in order to narrow down a macrocephaly locus. Of the genes described above, the most likely candidate gene appears to be* USP15* (see Table 2) because of its involvement in bone morphogenesis.

## Figures and Tables

**Table 1:** Comparison of the main clinical indications of Patients 1 and 2 and six previously reported patients carrying 12q14 microdeletions.

	Patient 1	Patient 2	Spengler et al. (2010) [3]	Takenouchi et al. (2012) [4]	Mari et al. (2009) [5]	Menten et al. (2007) [2] #D0502619	Menten et al. (2007) [2] #4818	Buysse et al. (2009) [1] #03g1858
Hg[19] coordinates	63,169,460–66,983,229	60,220,054–65,843,601	~66,200,000–67,550,000	66,080,229–70,062,135	~66,173,733–68,023,733	~61,800,000–66,800,000	~65,100,000–68,500,000	~61,500,000–67,600,000

Deletion size (Mb)	3.8	5.6	1.35	4	1.8	5	~3.4	~6

Pregnancy	IUGR	Uncomplicated	IUGR	Severe IUGR	Severe IUGR	Oligohydramnios	Hyperemesis	Uncomplicated

Birth measurements	Wgt.: 2550 g, Lgt.: 48 cm, HC: 35 cm	Wgt.: 2600 g (75th centile), HC: 33 cm (75th–91th centile)	Wgt.: 2700 g (−1.83 SD), Lgt.: 46 cm (−2.59 SD), HC: not noted	Wgt.: 527 g (−3.78 SD), Lgt.: 30.5 cm, HC: 22 cm	Wgt.: 1730 g (<10th centile), Lgt.: 43 cm (−4 SD), OFC: 29 cm (−3.66 SD)	Wt.: 2300 g (<P3), Lgt.: not noted, OFC: not noted	Wgt.: at 3rd centile, Lgt.: at 10th centile	Wgt.: 2060 g (<P3), Lgt. & HC not noted

Growth parameters	3 years: Wgt.: 11 kg (0.5 kg <3rd centile), Hgt.: 82.6 cm (−3.5 SD), HC: 49.5 cm (40th percentile)	3 years 8 months: Wgt.: 17.4 Kg, HC: 54 cm, Hgt.: 102.2 cm	1 year 9 months: Wgt.: 6.8 Kg (−5.4 SD), Hgt.: 70.8 cm (−4.5 SD), OFC: 43.7 (−3.3 SD)	29 months: Wgt.: 10.5 kg (−0.75 SD), Hgt. 76 cm (−3.13 SD), HC: 42.5 cm (−3.53 SD)	3 years: Wgt.: 6.5 Kg (−5 SD), Hgt.: 77 cm (−4.85 SD), OFC: 44.5 cm (−5.1 SD)	14 years: Wgt.: 51.3 kg (mean for age), Hgt.: 142.3 cm (−3.5 SD), HC: 53.3 cm (−0.66 SD)	18 years, Wgt.: 41 kg, Hgt.: 152 cm (both below 3rd centile)	16 years: Hgt. 131.5 cm (−6.2 SD), HC: 49 cm (−4.4 SD)

Clinical presentation	Severe SS, relative macrocephaly	Absolute macrocephaly	SS, FTT, relative macrocephaly	SS, severe FTT	Severe proportionate SS, FTT	SS	SS, FTT	Proportionate SS, FTT

Developmental delay	Mild	Yes	Mild	Not noted	Yes	Yes	Mild	Yes

Dysmorphic features	Alternating esotropia, severe myopia	Strabismus, prominent brow, large and slightly low set eyes	Prominent head, slight triangle face, dysplastic ears, clinodactyly of 5th finger	Cleft lip with alveolar cleft on the right, atrial septal defect	Triangle face with prominent forehead, low set ears, vaulted palate, micrognathia	Scoliosis, deep set eyes, bushy eyebrows, thin lips	Triangle face with wide spaced eyes	Synophrys, mild hypertelorism, broad & high nasal bridge, micrognathia & maxillary overbite

Behavioural issues	Possible ADHD, mild ASD	ASD	Not noted	No	Not noted	Not noted	Not noted	Not noted

HC: head circumference, OFC: Occipital Frontal Circumference, IUGR: Intrauterine Growth Retardation, ASD: Autism Spectrum Disorder, ADHD: Attention Deficit Hyperactivity Disorder, FTT: failure to thrive, and SS: short stature.

**Table 2:** OMIM genes that lie in the 12q14 interval bounded by *USP15* and *MSRB3*.

Gene	OMIM	Description
***USP15***	604731	USP15 is required for TGFB and BMP responses in both mammalian cells and frog embryos. The *USP15* gene encodes for a deubiquitinating enzyme. Animal models have shown that it regulates bone morphogenetic signalling during embryogenesis [10].

*PPM1H *	616016	Knockdown of PPM1H significantly increases proliferation in BT474 breast cancer cells [11].

*AVPR1A *	600821	The *AVPR1A* gene encodes for a receptor that is involved in vasopressin signalling, which is involved in behavioural responses, including stress management and territorial aggression as well as social bonding and recognition [12].

*DPY19L2 *	613893	*DPY19L2* represents the major gene causing globozoospermia [13].

*TMEM5 *	605862	The *TMEM5* gene encodes for a type II membrane protein of unknown function, but mutations have been associated with gonadal dysgenesis, neural tube defects, and most recently being a cause of severe cobblestone lissencephaly [14].

*SRGAP1 *	606523	SRGAP1 interacts with Roundabout transmembrane receptors, together with SLIT proteins, which guide neuronal and leukocyte migration [15].

*XPOT *	603180	Exportin-t is a specific mediator of tRNA export [16, 17].

*TBK1 *	604834	TBK1 is a binding partner for optineurin [18].

*RASSF3 *	607019	The *RASSF3* gene encodes for a putative tumor suppressor [19].

*GNS *	607664	The *GNS* gene encodes for N-acetylglucosamine-6-sulfatase, which is required for degradation of heparin sulphate. Homozygous mutations in this gene have been shown to be the cause of mucopolysaccharidosis type IIID [20].

*TBC1D30 *	615077	TBC1D30 protein is predicted to be involved in cell signalling [21].

*WIF1 *	605186	WIF1 binds to WNT proteins and inhibits their extracellular signaling involved in the control of embryonic development [22].

***LEMD3***	607844	*LEMD3* is involved in both BMP and TGF-beta signalling. Heterozygous loss-of-function mutations in the *LEMD3* gene are implicated in Osteopoikilosis and the associated Buschke-Ollendorff syndrome [8].

*MRSB3 *	613719	The *MSRB3* gene encodes for methioinine sulfoxide reductase. Homozygous mutations in this gene are associated with a form of autosomal recessive, nonsyndromic deafness [23].

Those genes in bold italics are implicated in bone morphogenesis.

TGFB: Transforming Growth Factor Beta.

BMP: Bone Morphogenetic Protein.
